# Simulated study on the grout-rock coupling mechanism and impermeability of chemical grouting materials in a single fracture

**DOI:** 10.1038/s41598-026-48950-0

**Published:** 2026-04-18

**Authors:** Ziwei Qian, Shenyang He

**Affiliations:** School of Resources and Geosciences, China University of Mining and Technology, Xuzhou, 221116 Jiangsu China

**Keywords:** Engineering, Materials science

## Abstract

Grouting in fractured rock masses is crucial for seepage prevention and reinforcement in underground engineering, yet the process remains poorly understood due to its concealment and complex grout-rock coupling. Using a self-developed visualization system, this study simulated grouting in a single planar fracture with four chemical grouts: acrylate, modified urea-formaldehyde, epoxy, and polyurethane. The entire process—including diffusion patterns, pressure evolution, fracture deformation, and post-curing seepage—was monitored, revealing the coupled seepage-deformation mechanisms during grouting, secondary migration, and stabilization. Under the grouting parameters used in this study, the results indicate that: under the 0.1 mm initial fracture aperture, polyurethane and acrylate diffuse best, epoxy worst. All grouts underwent secondary migration; polyurethane’s area expanded 160.7% (active expansion), others increased 34.3–77.6% (passive decay). Fracture grouting comprises injection, secondary migration, and stabilization stages. Impermeability varied: epoxy had high initial breakthrough pressure but dropped sharply; polyurethane showed adaptive cycles; modified urea-formaldehyde had high breakthrough but low stable pressure; acrylate had low peak pressure and reversible deformation but insufficient stiffness. This work provides a basis for material selection and process optimization.

## Introduction

Grouting, as a primary method for seepage prevention and reinforcement of fractured rock masses, is widely used in underground engineering. Understanding the migration and diffusion laws of grout within fractures is of significant guiding importance for grouting design and construction^1–3^. However, due to the concealed nature of the grouting process and the complex structure of fractured rock masses, current theoretical research on grouting in fractured rock lags behind practical engineering applications^4,5^. Furthermore, during fracture grouting, grout migration is governed by multiple interacting factors, including its own physico-chemical properties, injection pressure, fracture spatial distribution characteristics, roughness, and in-situ stress conditions. The significant coupling effects among these factors result in a highly complex grout diffusion process within fractures.

To investigate the laws governing grout migration and diffusion within fractures, scholars both domestically and internationally have conducted extensive research, primarily employing theoretical analysis^6–8^, numerical simulation^9–12^, and field tests^13,14^. However, theoretical analyses and numerical simulations are often based on simplified grouting methods and engineering conditions, leading to deviations between their conclusions and actual field conditions. Field tests, on the other hand, face difficulties in observing the real-time flow state of grout within fractures, making it challenging to obtain key parameters during the grout diffusion process. Furthermore, early theoretical models describing grout diffusion in fractures mostly assumed a constant fracture aperture during the grouting process^15–17^. In reality, the grout diffusion process results from the interaction between grout seepage and rock mass deformation. Although some studies have delved into the deformation behavior of fractured rock masses under grouting pressure^18–20^, they typically pre-defined the distribution pattern of grout pressure, thereby neglecting the feedback effect of fracture deformation on the grout diffusion process. Simultaneously, current research primarily focuses on the grouting behavior of a single or limited number of materials, lacking systematic comparison and mechanistic revelation of the complete grouting process in fractures for multiple categories of grouting materials under the coupling effect of grout seepage and rock deformation. Research on key stages, particularly from the secondary migration of grout in the later grouting phase to the formation of the consolidated grout body and the evolution of its impermeability characteristics, remains relatively limited. The filling and expansion of fractures by grout after injection is the fundamental mechanism by which high-pressure grouting generates additional stress in fractured rock masses. The seepage and diffusion condition of the grout within the fracture directly determines the sealing effectiveness and the impermeability performance of the grouted body.

To address the aforementioned shortcomings in existing research and fully account for the coupling effect between grout seepage and rock mass deformation during the grouting process in fractured rock, as well as the subsequent post-grouting stages, this study utilizes a self-developed visualized test system for grouting in a planar single fracture to conduct simulated experiments. This system enables real-time acquisition of grout pressure and multi-physical field information within the fracture, achieving transparent visualization of the entire grouting process. Based on this system, grouting tests in a planar single fracture were conducted using four chemical grouting materials commonly employed in engineering practice. The seepage and diffusion characteristics of different grouts during injection were systematically compared and analyzed. Furthermore, the entire process from the secondary migration of the unset grout—under the coupled action of grout pressure, fracture water pressure, and fracture stress after injection completion—until its final curing and solidification was investigated. Finally, seepage tests were performed on the consolidated grout bodies formed within the fracture to compare the effectiveness of the different grouting materials. This study reveals the differential mechanisms of various chemical grouting materials under the coupled seepage-deformation effects throughout the complete planar single-fracture grouting process, as well as the impermeability performance of their consolidated bodies. It provides important theoretical support for the selection of grouting materials and the optimization of process parameters in concealed environments, and offers experimental validation for existing theories on grout seepage-rock mass deformation coupling.

## Visualized grouting test in a single fracture

### Test apparatus

**Fig. 1:**
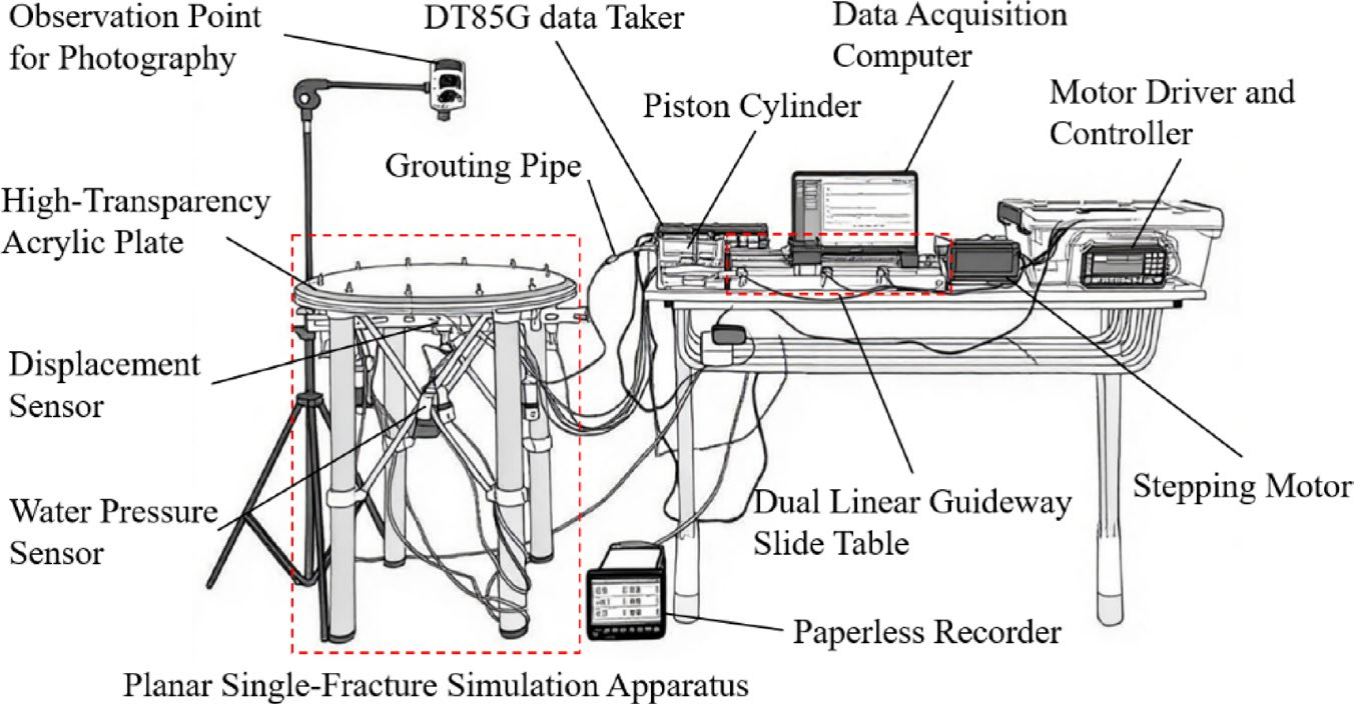
Visualized test system for grouting in a planar single fracture.

The tests in this paper were conducted using a self-designed visualized test system for grouting in a planar single fracture, as shown in Fig. 1. The system consists of a grouting control unit, a planar single-fracture simulation unit, and data acquisition and monitoring equipment. By simulating the coupling between grout seepage and rock mass deformation during the grouting process, the system can acquire real-time information on the internal physical field of the fracture and the deformation of the fracture plates. It can also simulate the grout flow state within a pressurized fracture under various influencing factors such as in-situ stress, fracture aperture, grouting flow rate, and grout mix ratio. The entire test apparatus enables dynamic visual monitoring of the complete grouting process, allowing for the simultaneous real-time monitoring and data acquisition of the grout diffusion path, fracture deformation, and pressure distribution.

To achieve visualized observation of the fundamental seepage process and quantitative analysis of fracture deformation, this study adopts key simplifications and assumptions regarding the actual fractured rock mass system: the naturally rough and morphologically complex fracture network is simplified into a single smooth planar fracture; the mechanical behavior of the actual rock on both sides of the fracture is simplified as a linear elastic model, simulated using acrylic material; and it is assumed that the grout, after being injected from the central borehole, propagates within the fracture in an axisymmetric, circular diffusion pattern. This simplified model successfully reveals the fundamental coupling mechanisms of grout seepage and rock deformation and partially reflects the hydromechanical coupling behavior of real rock masses. It quantitatively characterizes the fundamental relationships between grout pressure and the normal confining pressure acting on the fracture. It is important to note that, due to the presence of a three-dimensional in-situ stress field in actual fractured rock masses, shear dilation effects induced by fracture roughness, plastic damage in the surrounding rock, and fracture network connectivity, application to naturally complex fractures in engineering practice requires corrections based on geomechanical parameters.

The main body of the planar single-fracture simulation unit consists of two high-transparency acrylic plates, each 18 mm thick. These plates possess sufficient strength while also capable of undergoing certain elastic deformation under external forces. Their elastic modulus is significantly lower than that of real rock masses, which helps amplify deformation effects during simulated experiments. The purpose of this model is not to replicate the absolute deformation of specific rock types, but rather to clearly capture and quantitatively analyze the dynamic changes in fracture aperture under grouting pressure—that is, the coupling process itself. The edges of the two plates are connected and fixed by bolts, but a 0.1 mm aperture gap is maintained between the bolts. This gap is open to the atmosphere, serving as a fixed zero-pressure (atmospheric pressure) drainage boundary. This setup is used to simulate the condition where grout or water can freely discharge from this boundary. The unfolded diameter of the reserved grouting fracture space is 500 mm. Considering factors such as the test apparatus and the grouting medium, the initial fracture aperture in this study was set to 0.1 mm. On one side of the fracture plate, KTRC-type displacement sensors (range: 5 mm, accuracy: 0.001 mm) and MIK-P300-type pressure transmitters (range: 250 kPa, accuracy: 0.01 kPa) were installed. A total of five sensor modules were arranged at the center point of the plate and at radial distances of 50 mm, 100 mm, 150 mm, and 200 mm from the center along the radius. These are used to measure the deformation state of the fracture plate and the distribution of grout pressure within the fracture, effectively monitoring the overall condition of the preset fracture. The different measuring points are sequentially labeled as No. 1 to No. 5, in order of increasing distance from the grouting point.

**Fig. 2:**
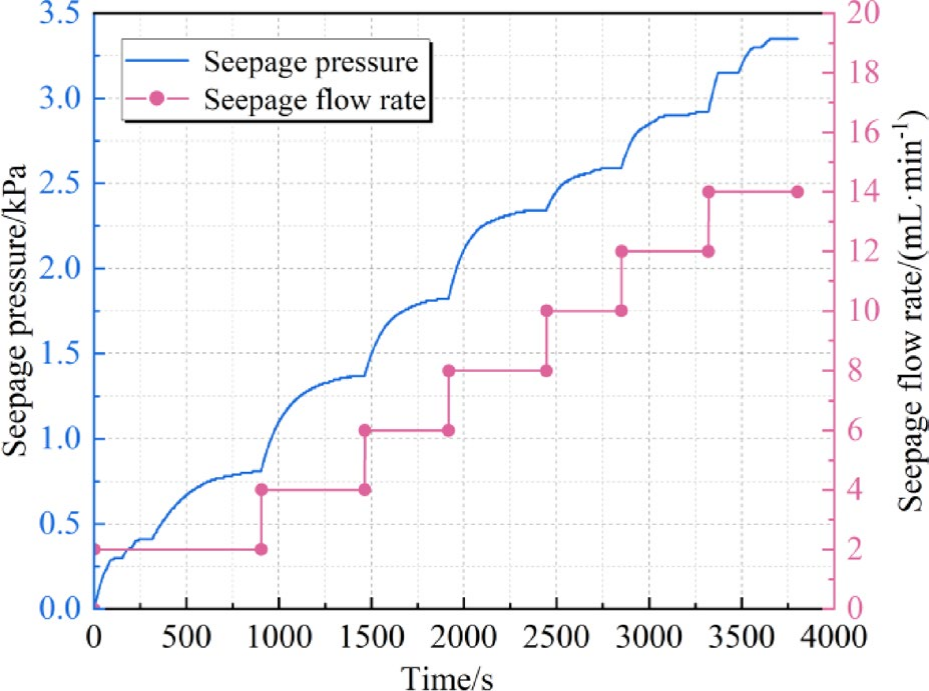
Temporal diagram of the dynamic response of flow rate and pressure during the water seepage process in the single fracture model.

**Fig. 3:**
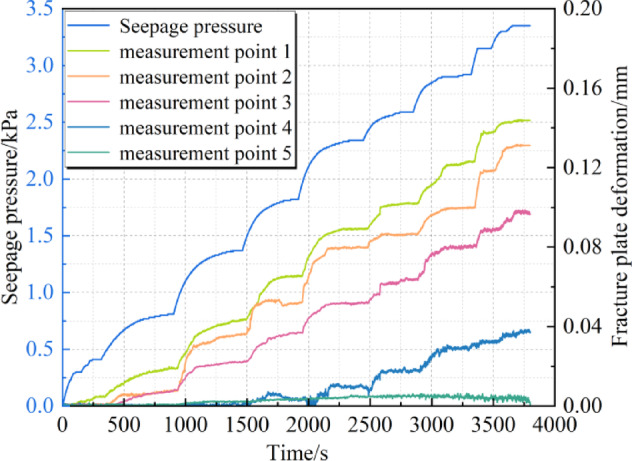
Temporal diagram of fracture plate deformation and dynamic pressure response during the water seepage process in the single fracture model.

To verify the reliability of the test system and understand the seepage characteristics of the planar single fracture, a water seepage test was conducted using the fully assembled apparatus. To ensure the accuracy of the test results, the interior of the fracture was first saturated with water before the seepage test began, and care was taken to prevent air ingress during the test. The seepage test employed multiple flow rate gradients, with a total of seven gradients set within a flow rate range of 2 to 14 mL/min. The seepage pressure was recorded at a frequency of 1 Hz. During the test, the system proceeded to the next flow rate gradient only after the seepage pressure had essentially stabilized at the current rate. The seepage test results for the model are shown in Figs. 2 and 3 below.

The test results from Figs. 2 and 3 indicate that during the water seepage process in the planar single fracture, the seepage pressure and fracture deformation exhibit significant flow rate-pressure coupling response characteristics and a spatial attenuation law. As shown in Fig. 2, the seepage pressure displays a typical stepped rising trend with the gradient increase in seepage flow rate. Furthermore, at each flow rate stage, the pressure rapidly stabilizes, indicating that the seepage field within the fracture reaches a dynamic equilibrium. This verifies the good reliability and stability of the test system. Figure 3 further reveals the response characteristics of fracture deformation during seepage: at different measurement points, the deformation shows a positive correlation with seepage pressure—higher pressure leads to greater changes in fracture aperture. Simultaneously, the deformation response exhibits clear spatial attenuation characteristics, manifested as smaller displacements at points farther from the water injection hole. This reflects the energy dissipation process during the transmission of seepage pressure within the fracture network. In summary, under these test conditions, the seepage behavior of the planar single-fracture model demonstrates the linkage where flow rate drives pressure and pressure induces deformation. The spatial attenuation law of its deformation response further reveals the synergistic interaction mechanism between pressure transmission and structural deformation during the fracture seepage process.

### Test materials

Currently, grouting technology can be categorized into two types based on the grouting materials: particulate grouts and chemical grouts^21–23^. Particulate grouts, represented by cement and clay, offer advantages such as low cost, simple preparation, and high consolidated strength, making them the most widely used grouting materials in engineering. However, traditional pure cement grouts have limitations, including long setting time, low consolidation rate, and poor grout stability, which restrict their application in areas like dynamic water sealing^24^. Chemical grouts primarily include silicate, acrylamide, lignin, epoxy resin, chrome lignin, urea-formaldehyde resin, polyurethane, etc., as well as cement-chemical composite grouts. They are characterized by low viscosity, good penetrability, and controllable gelation. For the tests in this study, four commonly used chemical grouting materials in engineering were selected: acrylate, modified urea-formaldehyde resin, epoxy resin, and polyurethane. A comparative analysis was conducted to investigate the seepage and diffusion characteristics, curing and formation process, fracture deformation during the complete planar single-fracture grouting process, and the impermeability of the resulting grout consolidation bodies for these different grouts.

These four chemical grouting materials have distinct characteristics, making them suitable for selection based on different ground conditions, construction techniques, and groundwater situations. Acrylate grout features low initial viscosity, enabling it to penetrate fine fractures; its gelation time is adjustable; it has a low permeability coefficient and strong compression resistance; and it is inexpensive. Its gel material is less hazardous, exhibits good stability, and can withstand wet-dry cycles. Its volume decreases in water-loss environments and rapidly expands when re-saturated, without damaging the gel structure during the process^25^. Modified urea-formaldehyde resin grout has relatively low viscosity, provides effective sealing and water-blocking, and possesses certain strength after curing, which can enhance the internal friction angle and cohesion within fractures, thereby improving ground stability^26^. Furthermore, its gelation time can be accurately adjusted according to project requirements to achieve optimal filling, and it can also be used for repair and water stoppage in underground dynamic water conditions. Epoxy resin grout offers excellent bonding performance, effectively filling cracks and pores in rock and soil to form a high-strength waterproof layer, thus addressing water leakage issues. Simultaneously, construction using epoxy resin is convenient, with fast curing speed, significantly improving water-blocking and anti-seepage effects^27^. Polyurethane material is an organic polymer composed of polyisocyanates and polyols, among other components. It possesses excellent bonding, sealing, chemical resistance, and injectability. It is widely used in construction, water conservancy, and transportation industries, primarily for crack repair, leakage prevention, and foundation reinforcement. It is also currently one of the most extensively used materials in coal mine water control^28–31^. A comprehensive performance comparison of these four chemical grouting materials is shown in Table 1 below.

**Table 1:** Comprehensive performance comparison of acrylate, modified urea-formaldehyde resin, epoxy resin, and polyurethane grouting materials.

Material	Acrylate	Modified urea-formaldehyde resin	Epoxy resin	Polyurethane
Main chemical components	Acrylate monomer, crosslinking agent, initiator, etc.	Urea-formaldehyde resin, curing agent, additives, etc.	Epoxy resin, curing agent, diluent, etc.	Polyurethane prepolymer, catalyst, foaming agent, crosslinking agent, etc.
Appearance & state	Water-soluble liquid with very low viscosity, can be diluted.	Water-soluble or solvent-based liquid with relatively low viscosity; solidifies into a brittle solid.	Two-component viscous liquid with high viscosity, requires mixing; solidifies into a high-strength solid.	Mostly two-component viscous liquid with high viscosity, requires mixing; solidifies into an elastic or semi-rigid foam.
Viscosity & injectability	Viscosity typically < 10 mPa·s; excellent injectability, can penetrate extremely fine cracks.	Viscosity at room temperature: 25–60 mPa·s; good injectability, can be injected into relatively fine cracks.	Viscosity: 200–1200 mPa·s (varies significantly with temperature); relatively poor injectability, usually requires dilution for finer cracks.	Viscosity generally: 100–2000 mPa·s; good injectability, can fill large voids after foaming.
Gelation/curing time	Within 1 h, adjustable from seconds to tens of minutes; good rapid-setting property.	Within 12 h, adjustable from 1 min to several hours by varying curing agent dosage.	Long curing time, typically several to tens of hours, but can be adjusted to fast-curing with curing agents.	Rapid reaction, gel time usually seconds to minutes; fast foaming and curing.
Consolidated body properties	Elastic gel with high toughness, water-swellable (effective for water sealing).	Rigid or semi-rigid gel, relatively high strength but brittle, non-elastic.	High-strength, high-bond-strength rigid solid with excellent mechanical properties; toughness can be improved with additives.	Elastic or semi-rigid foam with closed-cell structure, certain toughness.
Compressive strength	Relatively low (0.2–2.0 MPa); primary function is water sealing, not reinforcement.	Medium (5.0–30.0 MPa).	Very high (≥ 50 MPa); mainly used for structural repair and reinforcement.	Medium (2.0–20.0 MPa), adjustable based on formula; balances water sealing and certain structural strength.
Bonding strength	Good.	Relatively good.	Very high.	Excellent.
Durability	Good, but may shrink in long-term dry environments.	Relatively good durability.	Excellent, stable performance, long service life.	Good, water-resistant, chemically resistant, relatively good aging resistance.
Advantages & disadvantages	Advantages: Excellent permeability, controllable gel time, good flexibility, deformation-resistant. Disadvantages: Low strength, not for structural reinforcement, may shrink in dry environments.	Advantages: Relatively high early strength, good injectability, effective for dynamic water sealing. Disadvantages: Higher cost, may release formaldehyde, weak frost resistance.	Advantages: High mechanical strength, strong bonding, excellent durability. Disadvantages: Higher cost, limited injectability, strict construction requirements.	Advantages: Fast curing, water-swelling, effective water sealing, convenient construction. Disadvantages: Higher cost, moderate high-temperature stability, some products have slight irritating odor.

In this table, except for the parameters under “Appearance and Morphology”, “Viscosity and Injectability”, “Gelation/Curing Time”, and “Properties of the Consolidated Body”, which are based on experimental measurements or observations from this study, the remaining performance parameters are primarily compiled from the product technical data sheets of the relevant materials and the cited references^25–31^.

**Fig. 4:**
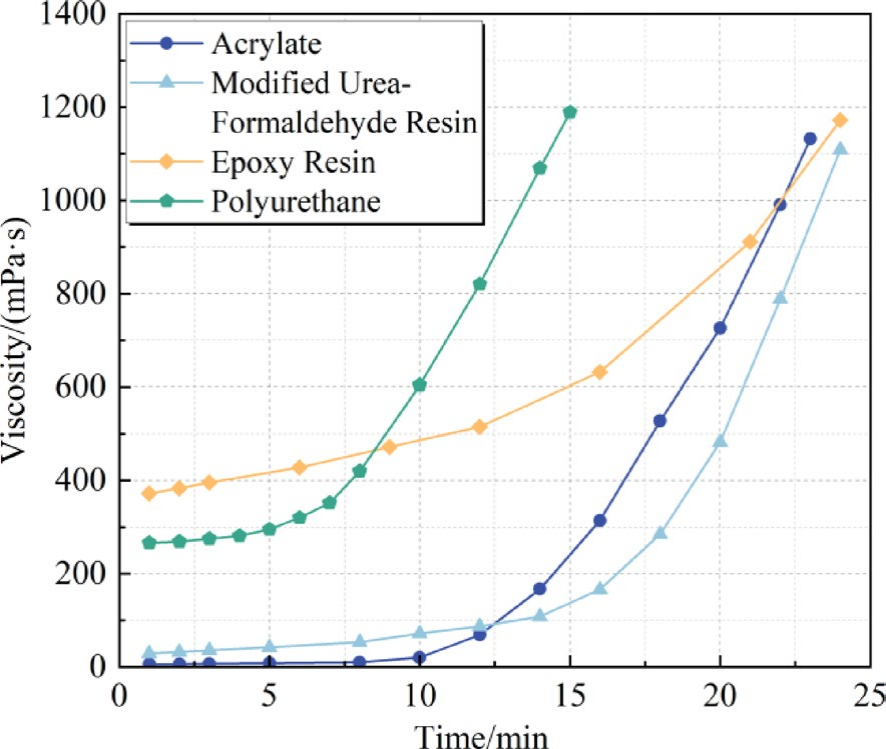
Temporal variation curves of viscosity for the four chemical grouting materials.

**Fig. 5:**
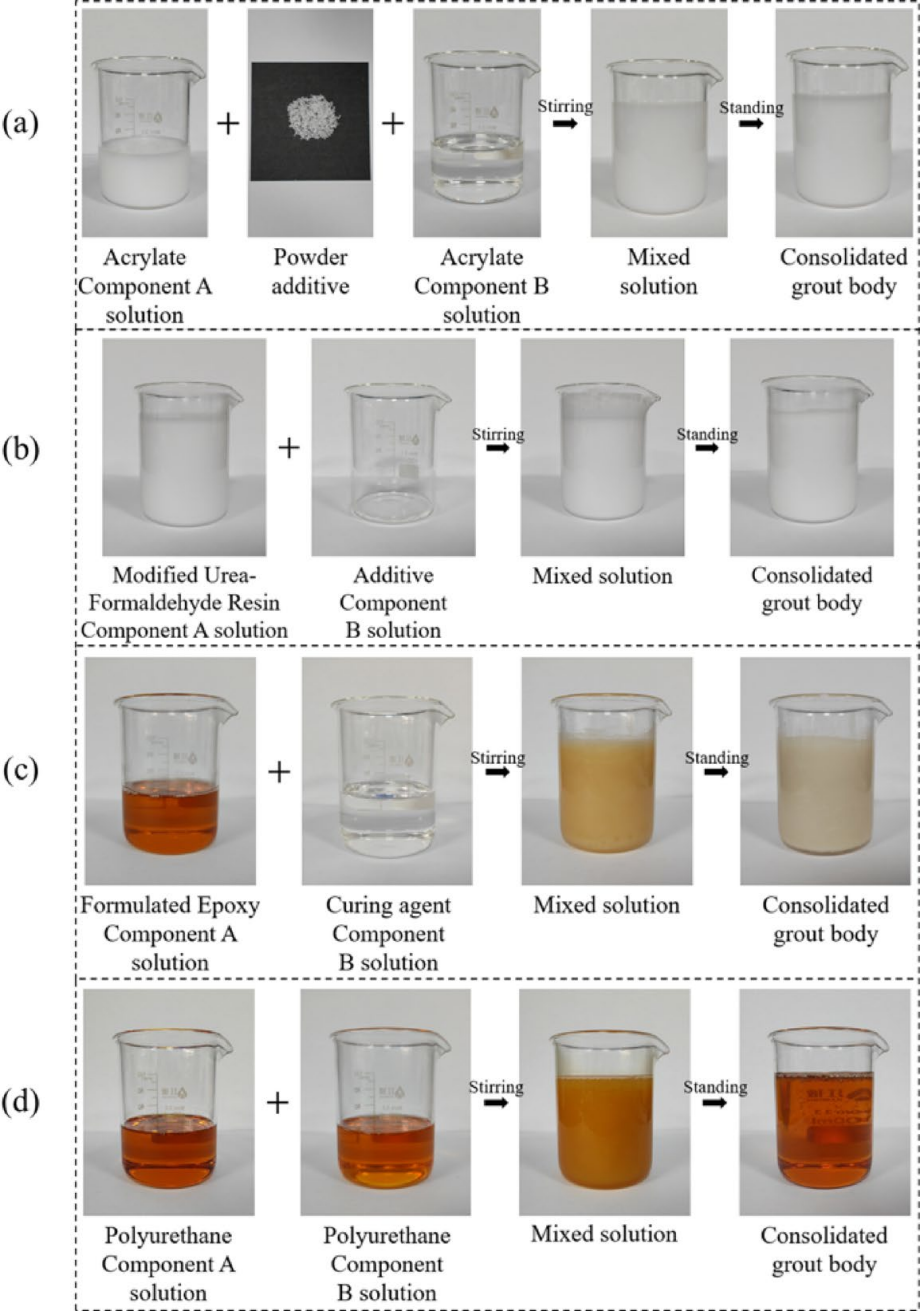
Morphology of the components, mixed solutions, and consolidated grout bodies for the four chemical grouting material formulations.

The four chemical grouting materials and their respective mix ratios used in the tests of this study are as follows: waterproof high-pressure acrylate grout (two-component type AB), comprising Component A solution, Component B solution, and a solid powder additive, with a mixing mass ratio = 1:1:0.001; modified urea-formaldehyde resin grout specialized for underground engineering in mining, comprising main liquid (Component A) and additive liquid (Component B), with a mixing mass ratio = 1:0.03; high-permeability modified epoxy resin grout (two-component type AB) specialized for mining grouting, comprising blended epoxy (Component A) and curing agent (Component B), with a mixing mass ratio = 1:1; and mining polyurethane grout (two-component type AB), comprising main agent (Component A) and reactant (Component B), with a mixing mass ratio = 1:1. After mixing and preparing the different grout materials according to their ratios, their rheological properties were measured under standard laboratory conditions with a digital viscometer. The curves of viscosity versus time for each grout are shown in Fig. 4. The components for the mix ratios of the aforementioned four chemical grouting materials, the mixed grouts, and the final morphology of the consolidated grout bodies are shown in Fig. 5.

### Test conditions

Under standard laboratory conditions, the grouts were prepared by mixing according to the aforementioned ratios. Subsequently, tests were conducted using the visualized test system for grouting in a planar single fracture. The injection hole in the fracture plate had a diameter of 8 mm, and the initial fracture aperture was set to 0.1 mm. The grouting flow rate for all chemical grouts was controlled at 10 mL/min, with a total grout volume of 30 mL injected for each test. Prior to commencing the grouting tests, the internal space of the fracture was fully saturated with water and allowed to stabilize for a period before grouting commenced. After the entire test and monitoring process was completed, and the grout had fully cured and stabilized, the grouting pipeline was cleared. A water seepage test was then performed on the consolidated grout body by injecting water through the original grouting hole. The water injection also employed multiple flow rate gradients, with seven different gradients set within a range of 2 to 14 mL/min. By acquiring the seepage pressure and fracture plate deformation data, the impermeability performance of the consolidated bodies formed by the different chemical grouting materials was compared and analyzed. Throughout the entire aforementioned test process, the interior of the fracture was maintained in a continuously water-saturated state to prevent the grouted body from drying out due to exposure to air, which could affect the accuracy of the results. This study aims to control other variables and, for the first time, compare the differences in the full-process behavior of the four chemical grouts under a specific initial aperture (0.1 mm). It should be noted that fracture aperture is a key variable affecting grout migration and sealing performance. The conclusions of this study are derived from a single aperture condition, and their quantitative relationships (e.g., diffusion area, pressure values) may change with variations in aperture.

## Test results and analysis

### Grout migration and diffusion patterns

**Fig. 6:**
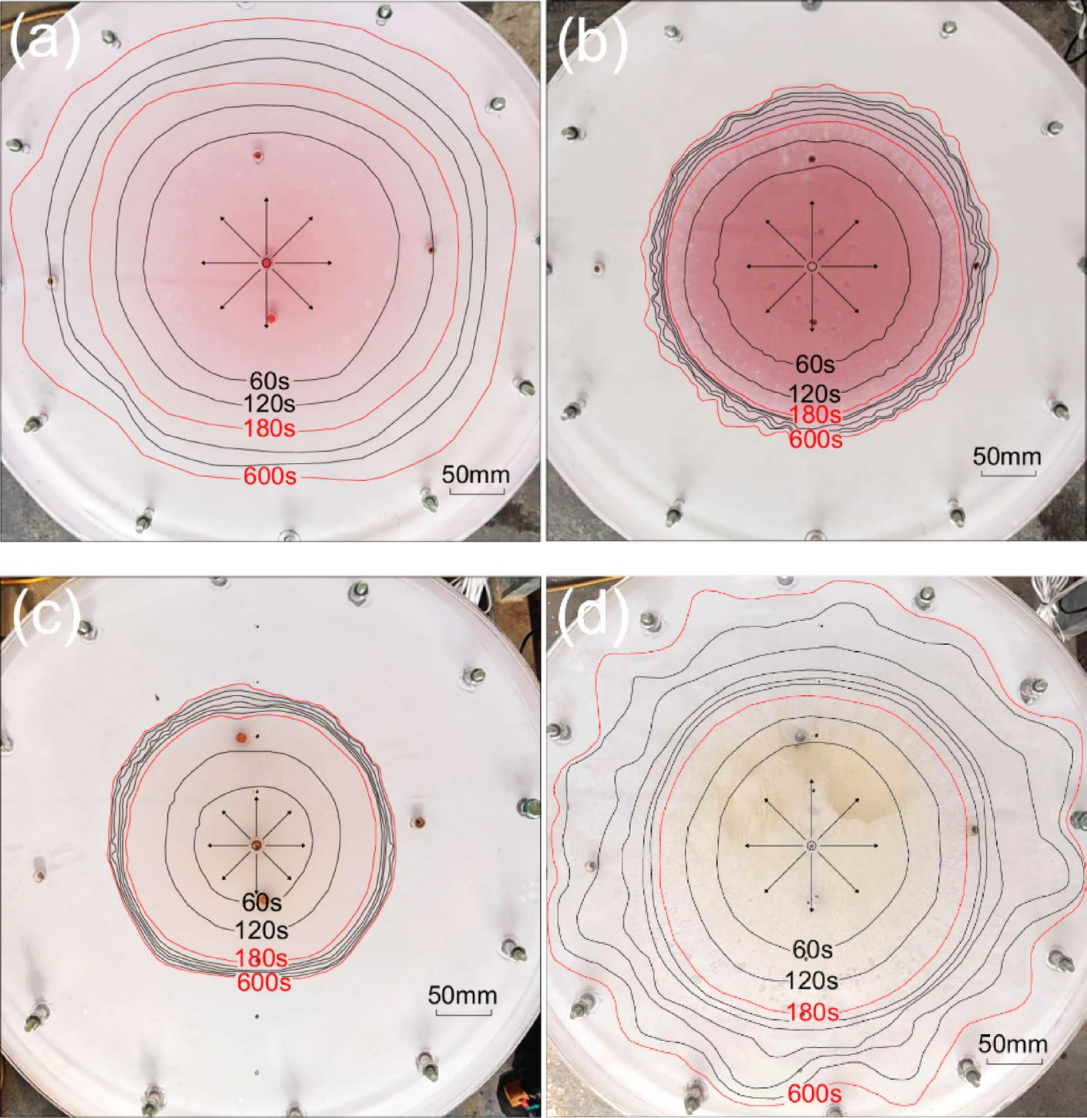
Migration and diffusion patterns of different grouts over time. (**a**) Acrylate, (**b**) Modified urea-formaldehyde resin, (**c**) Epoxy resin, (**d**) Polyurethane.

This study systematically investigated the migration and diffusion behavior of four types of chemical grouts—acrylate, modified urea-formaldehyde resin, epoxy resin, and polyurethane—within a planar single fracture. The migration and diffusion states of the different grouts throughout the entire grouting test were recorded by an image acquisition device mounted above the planar single-fracture simulation apparatus. The diffusion boundaries of the grout at different time points were quantitatively processed using image analysis techniques. These were then superimposed with the stabilized image of the grout on the observation interface, yielding the migration and diffusion patterns of the different chemical grouts throughout the test process, as shown in Fig. 6.

**Fig. 7:**
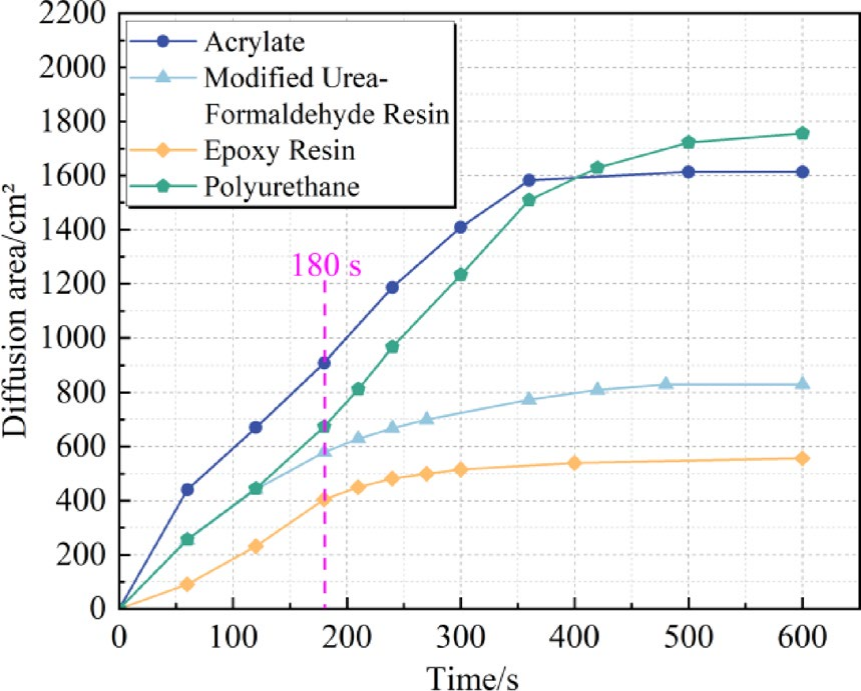
Diffusion area vs. time for the four chemical grouting materials.

During the entire test process, the period of 0–180 s corresponds to the active grouting stage. As shown in Fig. 6 above, all four chemical grouts exhibited a typical radial displacement diffusion pattern within the water-saturated fracture, with the diffusion morphology roughly forming a perfect circle centered on the grouting hole. At 180 s, when the predetermined grout volume was reached, the motor stopped advancing and remained in position, while monitoring continued after injection completion until full stabilization. It was observed that all grouts within the planar fracture continued to migrate and diffuse outward for a period after grouting stopped, differing only in the extent and duration of diffusion. During this stage, part of the grout within the fracture continued to seep and diffuse, driven by its own residual pressure gradient and the fluid-solid coupling effect caused by the deformation of the fracture plates. Furthermore, for polyurethane grouting materials, the chemical reaction involved is accompanied by a foaming process. The generated gas promotes volume expansion of the grout, creating significant foaming pressure, which in turn actively drives the grout to extend and diffuse into the surrounding areas of the fracture. Based on the captured images of the grout migration and diffusion patterns, the diffusion front was extracted through the following image-processing workflow: (a) The original images underwent grayscale conversion and Gaussian filtering for noise reduction; (b) Image binarization was performed using Otsu’s adaptive thresholding method to distinguish the grout region from the background; (c) The Canny edge detection algorithm was employed to identify the grout diffusion front, and discontinuous edges were connected via morphological closing operation; (d) Scale calibration was conducted by converting pixel coordinates to real-world physical coordinates using a calibration plate of known dimensions (the fracture model with a diameter of 500 mm) present in the images; (e) The number of pixels corresponding to the grout region in the binary image was calculated and converted to the actual area. Each image for a given time point was analyzed three times, and the relative error of the diffusion area was consistently less than 3%, indicating good repeatability of the image analysis method. The primary source of uncertainty in the experiment stems from local errors in edge detection, which were manually verified and controlled within 5%. This yielded curves showing the change in diffusion area over time for the four chemical grouts, as shown in Fig. 7 below.

Analyzing Fig. 7, under the grouting parameters used in this study, the diffusion characteristics of the different chemical grouting materials exhibit significant variations. These differences fully reflect the distinct features of each grout type in terms of rheological properties, curing mechanisms, and interaction with the fracture surfaces. Regarding the final diffusion morphology, the stabilized diffusion areas for the four chemical grouts—acrylate, modified urea-formaldehyde resin, epoxy resin, and polyurethane—after grouting completion were 1613.63 cm², 829.68 cm², 556.60 cm², and 1756.29 cm², respectively. These areas account for 82.18%, 42.26%, 28.35%, and 89.45% of the total fracture plate area (1963.50 cm²). A pronounced secondary migration phenomenon was observed for all four chemical grouts after injection stopped. Compared to their areas at the cessation of active grouting (180 s), the final diffusion ranges increased by 77.55%, 43.09%, 34.29%, and 160.70%, respectively. Based on a comprehensive comparative analysis, the acrylate grout demonstrated good diffusivity due to its low initial viscosity, with its diffusion area exceeding 80% of the total, indicating excellent permeability and low flow resistance. The polyurethane grout ultimately covered nearly 90% of the area. After injection, it rapidly underwent a chemical reaction, generating bubbles that caused expansion and diffusion, showcasing superior diffusion performance. The modified urea-formaldehyde resin grout showed moderate performance, exhibiting certain diffusion capability but relatively limited adaptability to the fracture medium. The epoxy resin grout had the smallest diffusion area, accounting for less than 30%, reflecting that its high viscosity and strong internal cohesion result in relatively weak migration and diffusion ability within the fracture.

### Grout pressure variation

**Fig. 8:**
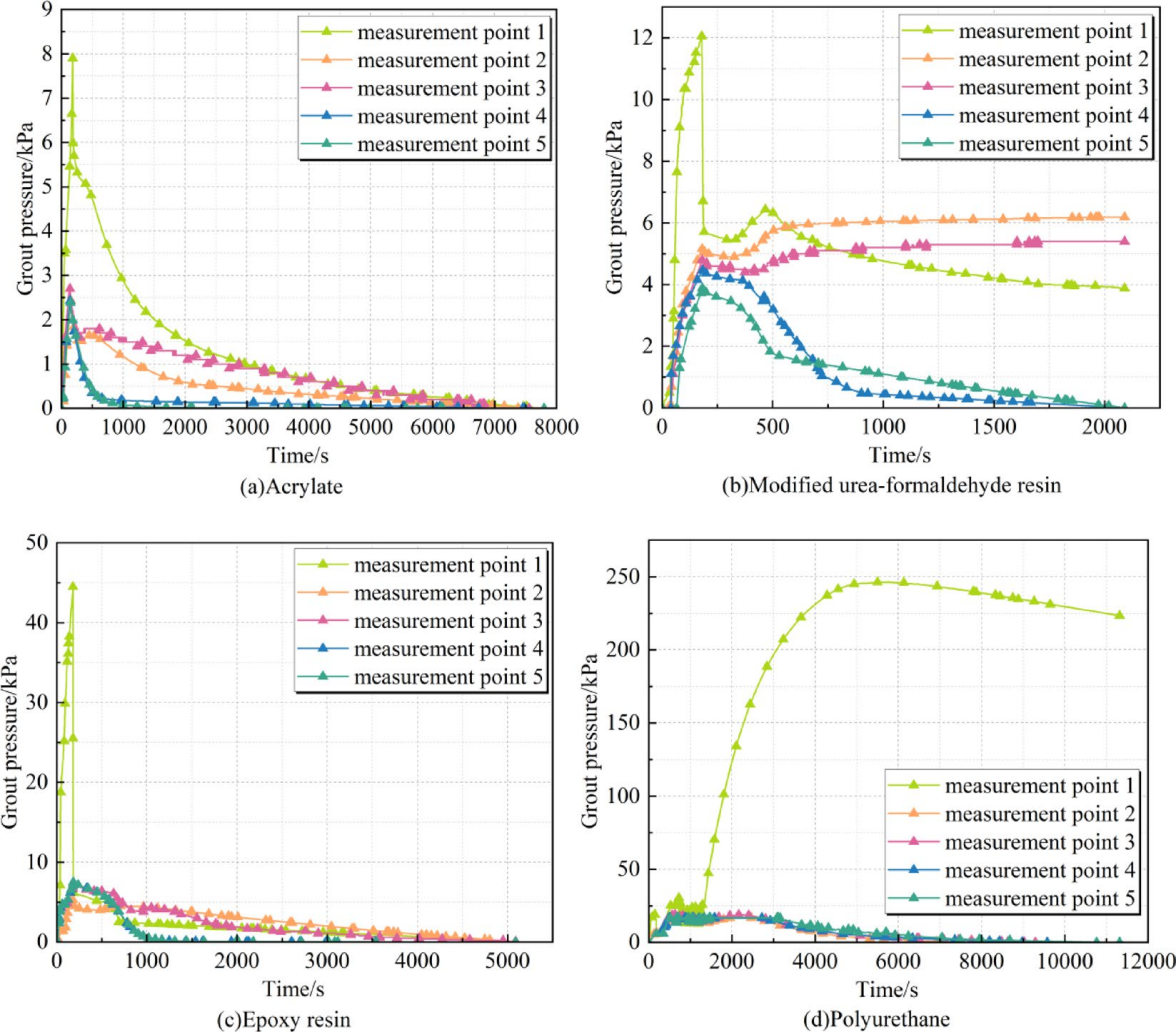
Grout pressure vs. time for the four chemical grouting materials.

This study utilized a pressure monitoring system installed on the fracture plate device to dynamically capture and quantitatively analyze the spatiotemporal evolution of the pressure field within the fracture throughout the entire grouting test. The test recorded the pressure versus time curves for the four different chemical grouts at various key measurement points within the fracture space, as shown in Fig. 8.

As shown in Fig. 8, under the grouting parameters used in this study, the temporal evolution curves of grout pressure for the four chemical grouting materials exhibit significant differences, reflecting their distinct rheological properties and curing mechanisms. The pressure curve for the acrylate grout shows relatively minor fluctuations and remains stable, with its peak pressure being much lower than that of the other materials. This aligns with its low viscosity and near-Newtonian fluid characteristics, where the grout has low flow resistance, and the grouting energy during the active injection stage is primarily converted into kinetic energy, resulting in a larger diffusion range. However, its lower injection pressure also implies a weaker fracturing and permeation effect on the fracture; sealing is mainly achieved through filling rather than compaction. The pressure of the modified urea-formaldehyde resin grout rises steadily during the active injection stage, indicating moderate viscosity and good fluidity. After grouting stops, the pressure decay process exhibits a certain degree of obstruction. This phenomenon stems from water molecules generated by the reaction and the formation of an early-stage gel structure, which increase the flow resistance. This time-dependent rheological property results in a moderate diffusion capability, ultimately forming a grouted body with relatively balanced performance. Furthermore, the setting time of this material can be adjusted according to the designed grouting range to achieve optimal filling. Due to its high initial viscosity, the pressure curve of the epoxy resin grout is characterized by a rapid surge in pressure at the injection hole shortly after grouting initiation, followed by a rapid decline after injection stops, while pressure changes at other monitoring points are relatively gentle. This indicates that the epoxy resin grout has strong internal cohesion, and flow must first overcome its own significant resistance, which directly explains its smallest diffusion radius. However, this high-pressure injection ensures tight compaction and penetration of the grout into the fracture walls, providing a foundation for its good bonding performance and impermeability. The pressure curve of the polyurethane grout is distinctly different. During the active injection stage, the pressure initially rises. After injection stops, the pressure remains stable for a period. Subsequently, due to rapid internal chemical reactions generating gas accompanied by volume expansion, significant foaming pressure is formed. This drives the grout to undergo noticeable secondary diffusion, and this pressure primarily acts near the injection center, causing a significant second pressure peak at the grouting hole. This characteristic allows it to achieve effective long-distance filling and dense compaction effects even after low-pressure injection, forming a composite grouted body with good mechanical properties.

### Characteristics of fracture plate deformation

**Fig. 9:**
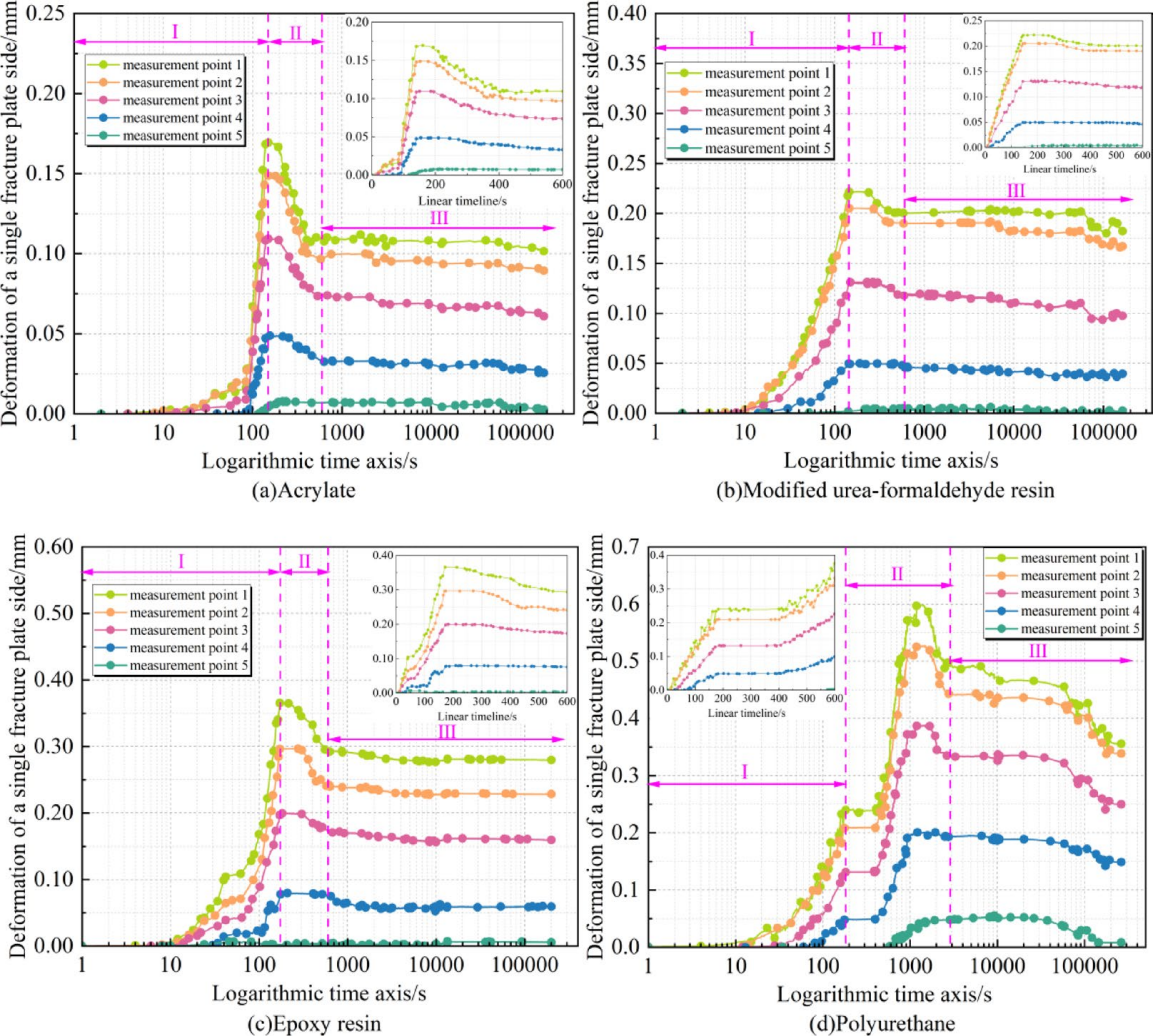
Deformation of a single fracture plate side during the entire grouting process for the four chemical grouting materials.

Throughout the entire grouting test process, this study employed a distributed displacement sensing system to conduct real-time monitoring of the deformation response at different radial positions on the surface of the fracture plate relative to the grouting hole. Given the strict symmetry of the upper and lower fracture plates in terms of geometric dimensions, material properties, and boundary constraints, monitoring the deformation of a single plate can accurately reflect the dynamic variation characteristics of the fracture aperture during grouting. Due to the long duration of the entire test, a logarithmic coordinate was applied to the time axis to effectively compress the large time span. This enhanced the visualization of the deformation data trend across all test stages and highlighted the details of fracture plate deformation changes during the initial grouting phase. The monitored results of the deformation of a single fracture plate over time are shown in Fig. 9. This data not only reveals the elastic deformation behavior of the fracture throughout the grouting test but also provides a key basis for analyzing the coupling mechanism between grout diffusion and fracture deformation.

Based on the test results shown in Fig. 9, the fracture deformation process induced by the four grouting materials—acrylate, modified urea-formaldehyde resin, epoxy resin, and polyurethane—can be divided into three typical stages: Stage I – Grouting Stage, Stage II – Grout Secondary Migration Stage, and Stage III – Grout Curing and Stabilization Stage. The underlying mechanisms for each stage are analyzed as follows:

Grouting Stage (Stage I): Under the active injection of these materials, the fracture deformation exhibited a rapid increasing trend. During this stage, the deformation is primarily dominated by the external grouting pressure, which overcomes the initial closure stress of the fracture, forcing the fracture walls to elastically open. Examining the details of the deformation evolution in the figure, the curves of fracture aperture versus time generally show a characteristic of “gentle at first, then steep.” This reflects the dynamic process of grout pressure establishment and propagation within the fracture. At the initial moment of grouting, the pressure first needs to overcome the initial closure stress of the fracture surfaces and the flow initiation resistance of the grout, gradually filling the area near the injection hole. At this time, the fracture opening rate is relatively slow. As grout injection continues, the pressure fully accumulates and propagates radially along the fracture, with the effective acting area continuously expanding, causing the fracture to enter a stage of rapid elastic opening, where the slope of the deformation curve increases significantly. Due to differences in rheological properties, the time of the inflection point from “gentle to steep” and the magnitude of the steep increase vary among the different grout materials, but all follow this fundamental mechanical process. Furthermore, the magnitude of fracture deformation is directly related to the peak grouting pressure and the grout flow resistance. Among them, the epoxy resin grout, due to its high initial viscosity resulting in higher pressure during the active grouting stage, caused the highest fracture plate deformation by the end of this stage. Polyurethane and modified urea-formaldehyde resin grouts, with their moderate viscosities and steady pressure rise, induced relatively intermediate levels of fracture plate deformation. In contrast, the acrylate grout, due to its lowest initial viscosity and good fluidity, generated the smallest grouting pressure under the same flow rate, consequently causing the relatively smallest fracture plate deformation.

Grout Secondary Migration Stage (Stage II): The fracture deformation behavior under different grouting materials showed significant differences. The fracture deformation induced by acrylate, modified urea-formaldehyde resin, and epoxy resin grouts began to decay. During this stage, the external driving force from grouting had ceased. The driving force for grout migration at this point shifted to the residual pressure within the system. This pressure dissipates rapidly during seepage. Simultaneously, the fracture plate rebounds under its own elastic restoring force, leading to a reduction in fracture deformation. Polyurethane grout exhibited unique behavior: its fracture deformation magnitude did not decrease after grouting stopped but instead increased significantly. This distinct phenomenon is directly attributed to its water-activated foaming chemical reaction, which generates gas accompanied by volume expansion, producing significant endogenous foaming pressure. This pressure effectively counteracts and surpasses the elastic recovery tendency of the fracture plates, thereby realizing an “active expansion after grouting completion” process. This is the core characteristic distinguishing polyurethane grout from other passive grouts.

Grout Curing and Stabilization Stage (Stage III): Over an observation period of approximately two days from the end of grouting until the grouted bodies were completely cured and stabilized, the fracture deformation for all grout material systems showed a certain degree of reduction. This phenomenon results from the combined action of various time-dependent mechanisms. For the acrylate grouted body, the three-dimensional network gel formed via free radical polymerization undergoes polymerization shrinkage during crosslinking, accompanied by the close arrangement of molecular chains and water migration^32^. For the modified urea-formaldehyde resin grout, chemical shrinkage during curing and physical shrinkage due to water loss are the primary factors. The cured epoxy resin body mainly exhibits polymerization and curing shrinkage^33–35^. The foam formed by the polyurethane reaction also experiences gas cooling contraction and intrinsic creep of the foam cell structure after complete curing^36–38^. The final stabilized value of fracture deformation in this stage comprehensively characterizes the effective mechanical support capacity provided to the fracture space by the consolidated bodies of various chemical grouts after completing their main reactions.

### Seepage test after fracture grouting

**Fig. 10:**
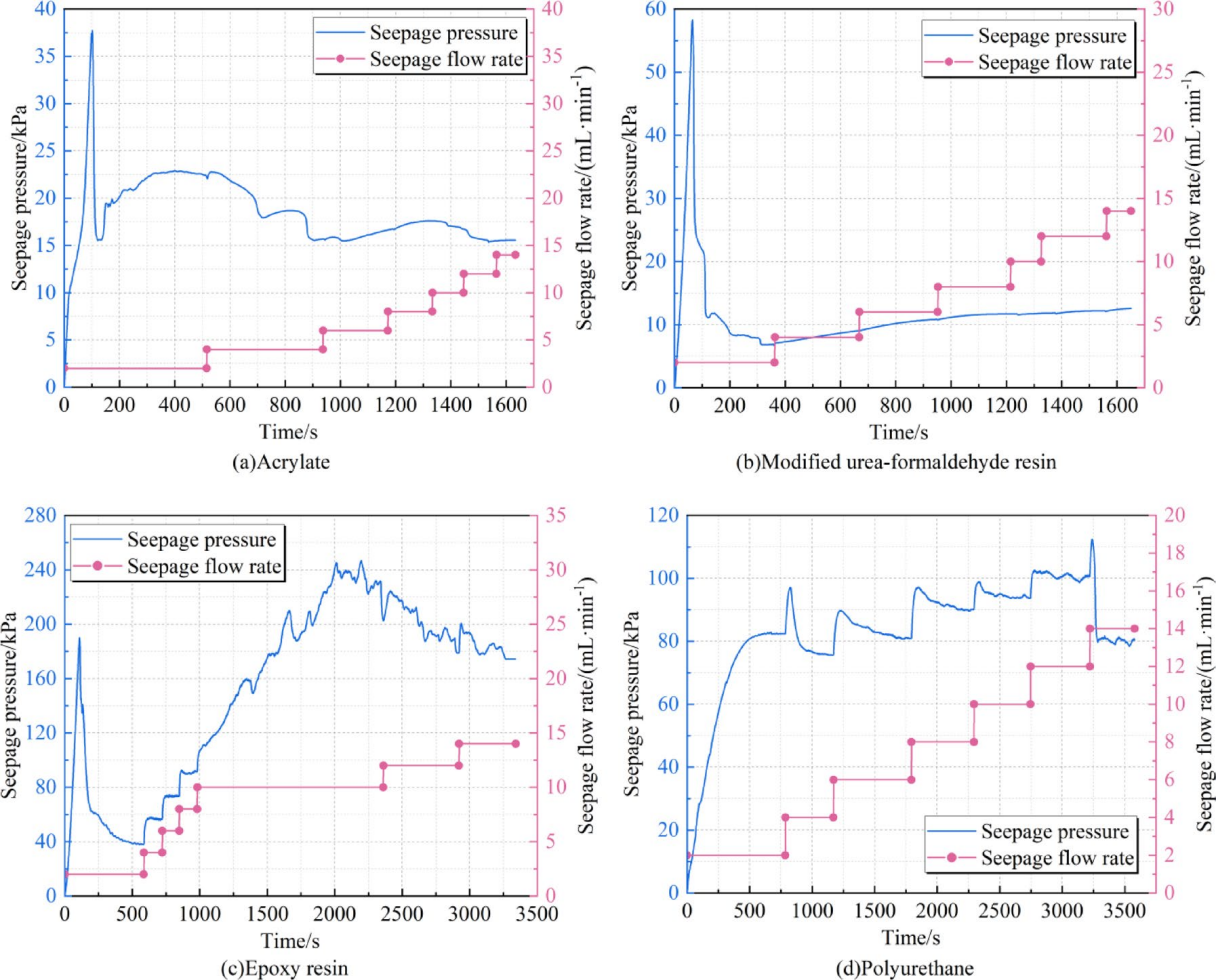
Seepage test results for the consolidated grout bodies of the four chemical grouting materials.

**Fig. 11:**
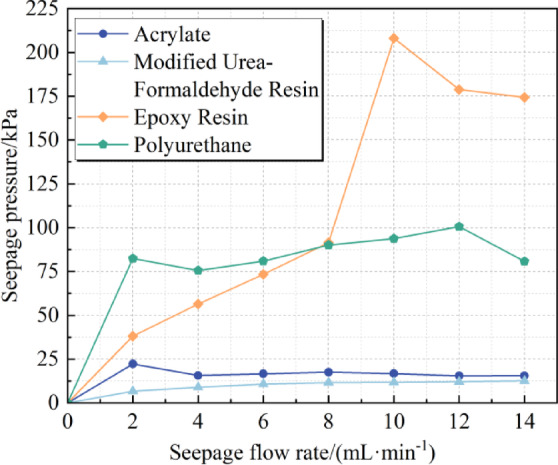
Relationship between seepage flow rate and seepage pressure for the consolidated grout bodies of the four chemical grouting materials.

After grouting was completed and the grout had fully cured to form a stable consolidated body within the fracture, the grouting pipeline was cleared. A seepage test was then conducted on the grouted fracture via the central injection hole to evaluate the impermeability performance and sealing effectiveness of the consolidated bodies formed by the different chemical grouting materials. This seepage test also employed multiple flow rate gradients, with seven gradients set within a range of 2 to 14 mL/min. During the seepage process, pressure transmitters were used to collect seepage pressure data, and displacement sensors were used to capture the deformation characteristics of the fracture plate, both at a sampling frequency of 1 Hz. During the test, the system proceeded to the next flow rate gradient only after the seepage pressure had essentially stabilized at the current rate, ensuring a continuous testing process. To distinguish whether seepage occurred through the consolidated grout body or along the grout-fracture interface, the seepage water was dyed, allowing for clearer identification of the seepage path at the interface. Figure 10 shows the seepage test results for the consolidated grout bodies of the four chemical materials, and Fig. 11 presents the relationship curves between seepage flow rate and seepage pressure for these bodies. These test results not only reflect the impermeability of the different grouting materials but also indirectly characterize the bonding strength between the grouted body and the fracture surface, as well as its durability, serving as an important basis for evaluating grouting material performance.

**Fig. 12:**
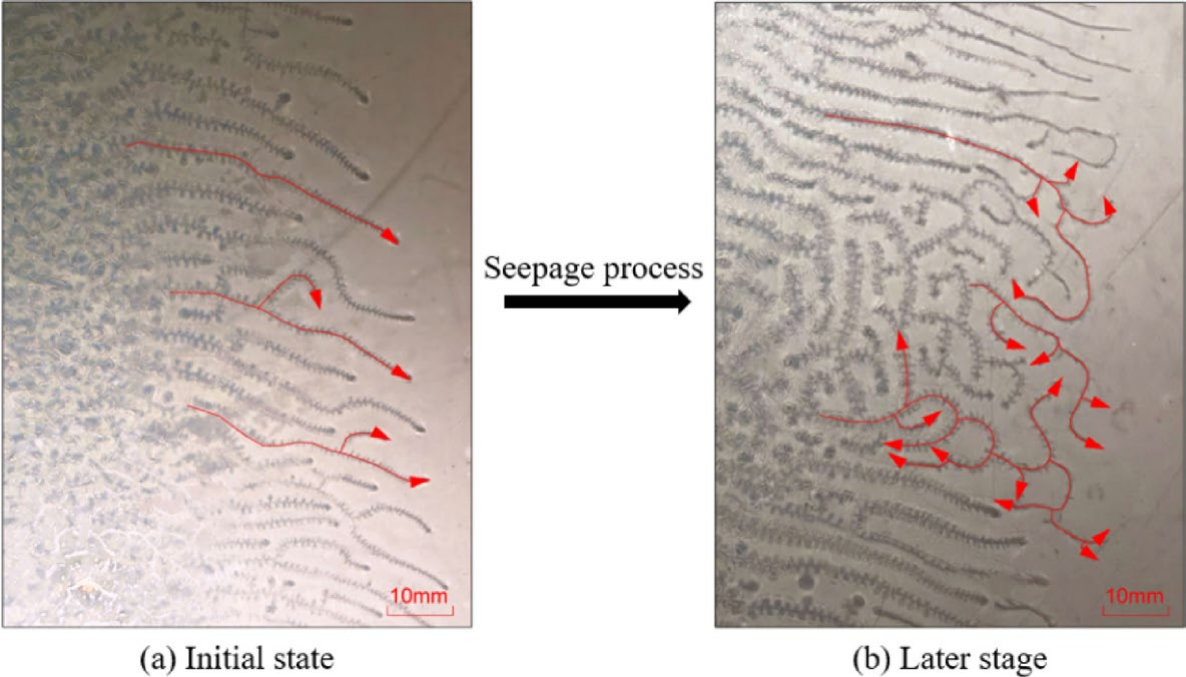
Comparison of microstructural evolution along the seepage path in the acrylate grouted body.

Based on the seepage test curve characteristics shown in Fig. 10, the impermeability performance and structural stability of the consolidated grout bodies formed by the four chemical materials can be analyzed as follows:

Acrylate Grouted Body: Its seepage pressure rapidly reaches an initial peak (37.71 kPa) and then quickly declines, followed by a gentle increase. This characteristic is closely related to the microstructural behavior of the hydrophilic polymer gel formed during its gelation and curing. The initial peak corresponds to the energy required to overcome the capillary resistance within the gel and the static friction at the interface when seepage initiates. The rapid pressure decline indicates that the gel structure undergoes elastic deformation and structural rearrangement under fluid shear, within the grouted body or at the interface forming initial seepage channels. The subsequent gentle increase may stem from the further, slow densification of the gel network during seepage, or minor adjustments at the gel-fracture interface under hydraulic action, leading to a slight increase in the tortuosity of the seepage path. This structural evolution process was clearly observable during the seepage test (Fig. 12). Overall, its cured body exhibits relatively high permeability. It primarily relies on the physical filling effect of the gel itself within the fracture, resulting in limited impermeability. However, the structure possesses good reversible deformation capability and adaptability.

Modified Urea-Formaldehyde Resin Grouted Body: It exhibits a relatively high initial breakthrough pressure (58.26 kPa), which rapidly declines after reaching the peak. Subsequently, during the following seepage stages, the pressure stabilizes overall at a relatively low plateau, showing only a slight, gentle increase as the seepage flow rate rises. This behavior corresponds to the dense, relatively rigid cross-linked network structure formed during its curing process. The high breakthrough pressure indicates a tight bond between the grouting material and the fracture surface, with effective microscopic filling, requiring significant energy to overcome the interfacial bonding force and structural resistance to initiate seepage. The rapid decline and sustained low pressure afterwards suggest the establishment of a stable initial seepage channel, which becomes the primary pathway for subsequent flow, allowing fluid to pass through relatively smoothly without continuously overcoming additional resistance. The gentle, minor pressure increase during seepage is primarily attributable to the increase in flow rate. The overall gentle pressure-flow rate relationship indicates that the cured body of this material forms a uniform and stable seepage impedance, demonstrating reliable medium-to-long-term impermeability.

Epoxy Resin Grouted Body: Throughout the seepage process, it exhibited distinctly different characteristics. Among the four materials, it showed the highest initial breakthrough pressure, reaching 190.00 kPa, demonstrating the high structural stiffness and excellent bonding strength to the fracture surface of its cured body, indicating good impermeability. As the seepage flow rate increased from 2 mL/min to 10 mL/min, the pressure continued to rise correspondingly, reaching a maximum of 246.80 kPa, consistent with the seepage behavior of a highly dense cured body. However, a critical turning point for judging its impermeability occurred when the flow rate was increased to 12–14 mL/min, where the pressure showed a significant drop (from 202.50 kPa to 174.41 kPa). This phenomenon suggests that under the continuously increasing seepage flow rate and pressure, “micro-crack initiation and propagation” within the grouted body or “local bond failure” at the grout-fracture interface may have occurred, leading to the formation of new dominant seepage channels, thereby reducing the overall seepage resistance. The overall seepage results indicate that the epoxy resin grouted body possesses superior impermeability, characterized by high intrinsic strength and better bonding to the fracture interface. However, it also carries a certain risk of degradation under excessively high seepage pressure.

Polyurethane Grouted Body: Its seepage response is the most distinctive, exhibiting a regular periodic pattern of “pressure rise – peak decline – stable plateau” at each flow rate increment. This is directly related to the mechanical behavior of its porous foam structure. With each increase in seepage flow rate, the fluid pressure first compresses the foam cells, causing a rapid rise in resistance (rising phase). As the cellular structure undergoes elastic or plastic adjustment, with some cell walls collapsing or flow channels widening, a new seepage equilibrium forms, leading to a subsequent decrease in resistance (declining phase). Finally, a stable pressure plateau, which rises with increasing flow rate, is reached (stable phase). This process demonstrates the ability of the polyurethane foam to dissipate fluid energy through the compressibility and adaptive deformation of its own structure. Ultimately, at the highest flow rate (14 mL/min), the pressure plateau decreased significantly (from 112.40 kPa to 80.35 kPa), indicating more substantial structural changes. However, unlike the other materials, it did not show a sudden pressure drop but maintained effective sealing through progressive structural adjustment, demonstrating a unique damage tolerance and an energy-dissipating impermeability mechanism.

Figure 11 illustrates the relationship between the seepage flow rate and the final stabilized seepage pressure for the consolidated grout bodies of the four chemical materials. The different grouting materials exhibit varied states under different seepage flow rates and pressures. In practical engineering, the selection can be made based on specific conditions such as the project’s hydrogeological setting.

**Fig. 13:**
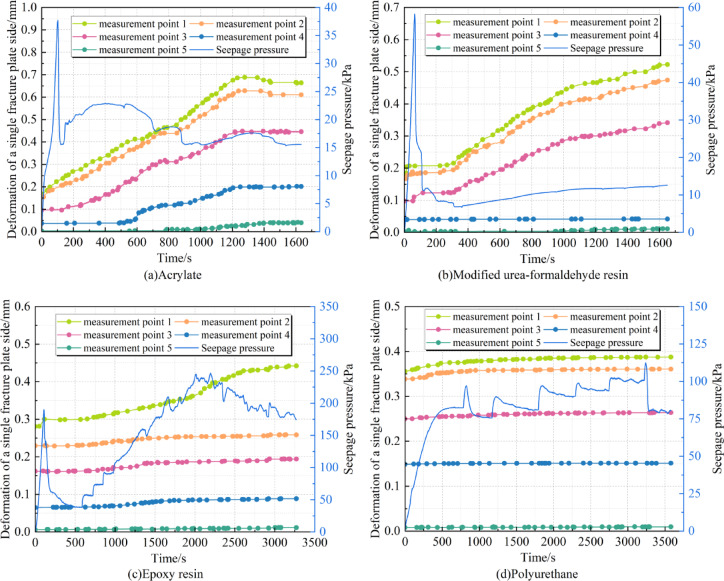
Seepage pressure vs. fracture plate deformation for the consolidated grout bodies of the four chemical grouting materials.

During the seepage test, to understand the deformation of the fracture plate, the deformation of a single fracture plate side was monitored and acquired via multiple displacement sensors arranged on its surface. Figure 13 below shows the curves of seepage pressure versus fracture plate deformation during the seepage process for the consolidated grout bodies of the four chemical grouting materials.

Based on the seepage pressure-fracture plate deformation relationship curves (Fig. 13) and the deformation response characteristics at different measurement points, the mechanical behavior and structural stability differences of the four chemical grouting materials under seepage action can be further revealed. The deformation behaviors and evolutionary patterns exhibited by the grouted bodies of different materials are highly coupled with the structural characteristics of their cured bodies and the aforementioned seepage mechanisms.

Acrylate Grouted Body: The deformation at all five measurement points continuously increased during the initial seepage stage. Subsequently, as the seepage pressure decreased steadily, the deformation rebounded synchronously and stabilized. This behavior aligns with the characteristics of its homogeneous and elastic hydrophilic polymer gel. Seepage pressure, transmitted through the fracture water, acts on the entire gel network and its interface, inducing overall, reversible elastic compressive deformation in the grouted body. The deformation correlates with the seepage flow rate and pressure, indicating that this material’s grouted body possesses good force-deformation coordination and elastic energy storage capacity under hydraulic loading. However, the relatively large overall deformation amplitude also reflects its comparatively low structural stiffness, suggesting a potential risk of cumulative deformation under long-term high water pressure.

Modified Urea-Formaldehyde Resin Grouted Body: The deformation at measurement points 1–3 (near the grouting hole) showed continuous growth, while the deformation at points 4 and 5 (edge regions) remained almost unchanged. This phenomenon is related to the seepage pressure characteristics observed in its seepage test—high initial breakthrough pressure but low stable pressure. The relatively high initial breakthrough pressure primarily acted on the region near the grouting hole, causing separation between the grouted body and the fracture wall in that area and forming the initial seepage channel, thus concentrating deformation there. Once the main seepage channel was established, the subsequent seepage pressure remained at a low level, insufficient to induce significant deformation in the edge regions. This reflects the high structural stiffness of its cured body, where both deformation and seepage are concentrated near the established dominant channel, resulting in good overall stability but limited deformation coordination.

Epoxy Resin Grouted Body: The deformation response occurred primarily at measurement point 1 (the grouting hole location). Its increasing trend generally aligned with the changes in seepage pressure, while the deformation increments at the remaining points were relatively small. This directly corresponds to its high-stiffness, high-bond-strength curing characteristics. Under high seepage pressure, the grout body itself undergoes minimal compressive deformation. However, as the initial seepage pressure primarily acts on the region near the injection hole, it is sufficient to overcome the local interfacial bonding force, causing a slight, channelized opening at the grout-fracture interface. When the seepage pressure increases, this microscopic channel is further opened, manifesting as an increase in fracture plate deformation, which also leads to the later drop in seepage pressure and a consequent slowdown in deformation growth. The fracture plate deformation pattern indicates that the seepage pressure is mainly dissipated in opening and maintaining a fine interfacial seepage channel, rather than causing overall failure of the grout body. Due to the material’s high stiffness and strong adhesion, it demonstrates excellent impermeability.

Polyurethane Grouted Body: During the initial seepage stage, measurement points 1–3 (near the grouting hole) showed only minor increases in deformation before stabilizing, while points 4 and 5 (edge regions) exhibited almost no change. This overall stable deformation state corresponds to the periodic adaptive behavior of its porous foam structure under seepage, characterized by the “pressure rise – decline – stabilization” pattern observed in the seepage pressure response. Under hydraulic action, the foam cells adapt to pressure changes through rapid elastic compression and structural reorganization. Deformation primarily occurs during the structural optimization and adjustment phase and is mainly confined to the region near the grouting hole where the dominant seepage path develops. After the structure completes its adaptive adjustment, the overall deformation of the fracture plate tends to stabilize even as seepage pressure continues to change, demonstrating excellent deformation self-limiting capability and structural stability. This indicates that the polyurethane grouted body can adapt to seepage pressure through localized structural adjustments, thereby avoiding significant overall deformation or damage propagation.

Deformation response during seepage is a key indicator for evaluating the long-term performance and failure modes of grouted bodies. The acrylate grouted body is suitable for low-stress environments that permit relatively large elastic deformation. The modified urea-formaldehyde resin is applicable to fractures with moderate pressure requiring local stability and controlled deformation. The epoxy resin is suited for conditions with high static water pressure but where strict prevention of pressure fluctuations and peak overloads is necessary. Polyurethane is appropriate for scenarios with dynamic water flow where the material must possess certain impermeability. This analysis of deformation characteristics, coupled with the seepage behavior, also serves as an important basis for the selection and design of grouting parameters.

## Discussion

This study systematically revealed the full-process behavior of four chemical grouting materials within a planar single fracture under the grouting parameters employed, utilizing a self-developed visualized experimental system. These findings hold clear significance for deepening the theoretical understanding of fracture grouting. Existing theoretical models often treat fractures as rigid boundaries[15–17], or assume pressure distributions while neglecting the feedback of fracture deformation on grout diffusion[18–20]. Moreover, they lack systematic comparisons for multiple materials, especially concerning secondary grout migration in the later stages and the behavior of consolidated bodies. The experimental results of this study overcome these limitations in previous research.

Firstly, through real-time monitoring and data acquisition, this study intuitively verified and quantified the dynamic coupling process between grout seepage and simulated fracture deformation. The experiments demonstrated that fracture aperture continuously changes throughout the entire grouting cycle and can be divided into three typical stages based on grout characteristics, which breaks away from the classic “fixed fracture aperture” hypothesis. In particular, Polyurethane grout, due to its foaming reaction, continued to drive active fracture expansion after grouting cessation, while other materials exhibited passive decay. This difference highlights the key role of the grout’s inherent chemical reaction mechanisms in the coupling process, serving as an important supplement to existing models that primarily focus on rheological coupling.

Secondly, this study clearly distinguished and explained the differential mechanisms of “secondary migration.” All grouts continued to diffuse after active injection ceased, but the “active expansion” type of Polyurethane is fundamentally different from the “passive decay” type of other materials. This deepens the understanding of the formation mechanism of the final grout diffusion range, indicating that it is not only determined by injection parameters but is also significantly influenced by the grout’s curing kinetics. Simultaneously, by incorporating fracture deformation during the curing shrinkage stage, the study presented the complete process of fracture grouting from fluid injection to consolidated body formation, addressing the previous lack of focus on the later stages of grouting.

Finally, from the perspective of “seepage-deformation” coupling, this study provided a new mechanistic basis for evaluating the impermeability of grouting materials. The experiments not only measured seepage pressures but also revealed the failure modes of each material’s consolidated body under multi-gradient seepage flows: Epoxy Resin’s high breakthrough pressure is accompanied by the risk of sudden degradation under high seepage pressure; Polyurethane achieves adaptive energy dissipation through the periodic compression-rebound of its foam structure; Acrylate exhibits good deformation coordination but limited strength; Modified Urea-Formaldehyde Resin tends to form stable dominant seepage channels. These findings, based on the structural response essence of the materials, provide richer experimental evidence for selecting grouting materials tailored to different hydrogeological conditions.

The homogeneous, smooth, elastic acrylic plate fracture model adopted in this study can effectively reveal the fundamental sequence and mechanisms of coupling between grout seepage and fracture deformation (e.g., pressure-driven opening, elastic rebound, curing shrinkage). However, it cannot simulate the heterogeneity, plastic deformation, joint surface mechanical behavior, or in-situ stress fields of real rock masses. Therefore, the experimental data obtained from the model do not possess direct engineering conversion significance. Nevertheless, the revealed stage-wise characteristics of the coupling process and the differences in deformation patterns induced by different grouts provide important reference value for understanding the mechanical mechanisms of on-site grouting.

In summary, this study, through simulated fracture grouting visualization experiments, provided direct evidence for grout-rock coupling theory and emphasized the critical role of material curing mechanisms; it systematically elaborated on the active and passive mechanisms of secondary migration for chemical grouts, refining the understanding of the full process of grouting effectiveness formation; and from the perspectives of seepage stability and structural failure modes, it deepened the understanding of the overall impermeability performance differences among different chemical grouting materials in fracture systems. The three-stage evolution model of the full grouting process, derived based on this, provides a new framework for a more comprehensive analysis of the interrelationships among grout diffusion, pressure evolution, fracture deformation, and final impermeability. However, the conclusions of this study are based on an idealized planar single-fracture model with a specific initial aperture. Complex factors in practical engineering, such as multiple apertures, rough fractures, network systems, and in-situ stress fields, are directions that require in-depth exploration in subsequent research.

## Conclusions

1. Under the 0.1 mm initial fracture aperture, polyurethane and acrylate exhibited optimal diffusion capabilities within the water-saturated fracture, with their final diffusion areas accounting for 89.45% and 82.18% of the total area, respectively. The diffusion of epoxy resin was limited by its high viscosity, reaching only 28.35%. Secondary migration occurred in all grouts after injection. The diffusion area of polyurethane increased by 160.70% compared to its area at injection cessation due to foaming reaction, classifying it as an active expansion type. The increases for acrylate, modified urea-formaldehyde resin, and epoxy resin were 77.55%, 43.09%, and 34.29%, respectively, classifying them as passive-decay types.
2. The evolution of grout pressure was closely related to material properties. Acrylate pressure was low and stable, conforming to Newtonian fluid behavior. Modified urea-formaldehyde resin pressure rose steadily, and its decay was hindered after grouting, reflecting its time-dependent rheological response. The pressure at the epoxy resin injection hole surged rapidly and then declined quickly, indicating strong internal cohesion. Polyurethane exhibited a unique secondary pressure peak, originating from endogenous foaming pressure driving secondary diffusion and active fracture expansion.
3. Based on the dynamic response characteristics of fracture plate deformation and grout diffusion behavior throughout the entire process, the grouting process was divided into a three-stage evolutionary model. In the grouting stage, deformation was dominated by active injection pressure. In the secondary migration stage, deformation for acrylate, modified urea-formaldehyde resin, and epoxy resin decayed due to residual pressure dissipation, while polyurethane showed counter-trend growth driven by foaming pressure, both leading to further expansion of the grout diffusion range. In the curing and stabilization stage, all materials exhibited varying degrees of curing shrinkage.
4. The impermeability of the consolidated grout bodies showed material-specific patterns. Under the grouting parameters used in this study, epoxy resin had the highest initial breakthrough pressure (190.00 kPa), but a sudden pressure drop occurred at the high seepage pressure stage, indicating potential degradation risk under high pressure. Polyurethane showed a periodic “pressure rise – decline – stabilization” response, reflecting the energy-dissipating adaptive mechanism of its foam structure. Modified urea-formaldehyde resin had a relatively high breakthrough pressure (58.26 kPa) but low stable pressure, with deformation concentrated near the grouting hole. Acrylate had a low peak pressure and overall reversible deformation, reflecting the characteristics of an elastic gel but with insufficient stiffness.
5. Under the idealized planar single-fracture laboratory conditions of this study, the four chemical grouting materials exhibited differentiated performance characteristics, providing preliminary experimental insights for material selection in fractured rock mass grouting. Polyurethane, with its foaming-driven active secondary diffusion and adaptive impermeability features, offers an experimental reference for the rapid sealing of fractures with flowing water. Acrylate, due to its reversible deformation properties, is suitable for water-stopping operations in low-stress environments. Modified Urea-Formaldehyde Resin, with its high initial breakthrough pressure and excellent macroscopic impermeability, provides an experimental reference for the seepage prevention and reinforcement of fractures under high water head. Epoxy Resin is applicable for grouting and water plugging in medium-pressure environments. It must be emphasized that the above analysis and insights are derived from a specific, idealized laboratory model. In practical engineering, grout diffusion behavior and the final sealing effectiveness are influenced by multiple interacting factors. Material selection must also involve comprehensive decision-making based on engineering costs, environmental requirements, construction conditions, and other relevant considerations.

## Data Availability

The datasets generated and/or analysed during the current study are not publicly available due to the confidential nature of the participant data and the terms of consent agreed upon, but are available from the corresponding author on reasonable request.
